# Optimization Method to Predict Optimal Noise Reduction Parameters for the Non-Local Means Algorithm Based on the Scintillator Thickness in Radiography

**DOI:** 10.3390/s23249803

**Published:** 2023-12-13

**Authors:** Bo Kyung Cha, Kyeong-Hee Lee, Youngjin Lee, Kyuseok Kim

**Affiliations:** 1 Precision Medical Device Research Center, Korea Electrotechnology Research Institute (KERI), 111 Hanggaul-ro, Sangnok-gu, Ansan-si 15588, Republic of Korea; bkcha@keri.re.kr (B.K.C.); khlee@keri.re.kr (K.-H.L.); 2 Department of Radiological Science, Gachon University, 191 Hambangmoe-ro, Yeonsu-gu, Incheon 21936, Republic of Korea; 3 Department of Biomedical Engineering, Eulji University, 553 Sanseong-daero, Sujeong-gu, Seongnam-si 13135, Republic of Korea

**Keywords:** noise reduction, parameter optimization, scintillator thickness, image restoration, non-local means (NLM) method, image quality assessment

## Abstract

The resulting image obtained from an X-ray imaging system depends significantly on the characteristics of the detector. In particular, when an X-ray image is acquired by thinning the detector, a relatively large amount of noise inevitably occurs. In addition, when a thick detector is used to reduce noise in X-ray images, blurring increases and the ability to distinguish target areas deteriorates. In this study, we aimed to derive the optimal X-ray image quality by deriving the optimal noise reduction parameters based on the non-local means (NLM) algorithm. The detectors used were of two thicknesses (96 and 140 μm), and images were acquired based on the IEC 62220-1-1:2015 RQA-5 protocol. The optimal parameters were derived by calculating the edge preservation index and signal-to-noise ratio according to the sigma value of the NLM algorithm. As a result, a sigma value of the optimized NLM algorithm (0.01) was derived, and this algorithm was applied to a relatively thin X-ray detector system to obtain appropriate noise level and spatial resolution data. The no-reference-based blind/referenceless image spatial quality evaluator value, which analyzes the overall image quality, was best when using the proposed method. In conclusion, we propose an optimized NLM algorithm based on a new method that can overcome the noise amplification problem in thin X-ray detector systems and is expected to be applied in various photon imaging fields in the future.

## 1. Introduction

Radiological imaging systems have established themselves as the most representative method among various imaging systems (e.g., optics and infrared), using the strength of the noninvasive identification of internal structures [1]. According to the Beer–Lambert Law, radiation is attenuated to energy depending on the linear attenuation coefficient and transmitted length of the material according to the energy of the radiation. The attenuated signal is then detected, which provides visual information [2]. However, various distortions occur owing to the interaction of radiation with the material, one of which is noise generation owing to a lack of photons [3]. Fundamentally, the radiation emission and attenuation processes are random and can be defined as Equation (1):

(1) $$P(n,\lambda )=\frac{e^{-\lambda }\lambda^{n}}{n!},$$

where λ is the mean value of independent trials and n is the outcome, which must be a positive integer. If $\bar{q}$ is the mean number of quanta, the standard deviation is equal to the $\sqrt{\bar{q}}$. Noise lowers the outline and contrast of objects in the image, making the boundary unclear, which creates side effects that lower detection capabilities from a visual perspective [4]. The Rose model demonstrates that the performance of an imaging device can be evaluated at the absolute scale (quantum efficiency) using the signal-to-noise ratio (SNR) model. This is of great significance because it lays the framework for research on the ultimate limitations of noise effects and sensitivity and can evaluate the correlation between the performance of electronic devices and human visual systems on a quantitative scale [5]. In an imaging system using a coupled charge-coupled device (CCD), the SNR considering quantum noise and electrical noise is defined as Equation (2) [6].

(2) $${SNR}_{value}=\frac{C\sqrt{\eta N_{i}}}{\sqrt{\left(1+\frac{1}{g_{1}g_{2}}+\frac{1}{g_{1}g_{2}g_{3}}\right)}+\frac{N_{a}^{2}}{{\left(g_{1}g_{2}g_{3}\right)}^{2}\eta N_{i}}},$$

where C is subject contrast, η is the quantum efficiency of the intensifying screen or scintillator, $N_{i}$ presents the X-ray photon flux, and $N_{a}$ is the total additive noise. $g_{1}$, $g_{2}$, and $g_{3}$ are the conversion ratios between X-rays and light, the optical coupling efficiency, and the quantum efficiency of the CCD or other electronic detectors, respectively [7]. Detective quantum efficiency (DQE) was defined as follows:

(3) $${DQE}_{value}(f)=\frac{{SNR}_{out}^{2}(f)}{{SNR}_{in}^{2}(f)},$$

In Equation (3), f denotes spatial frequency, ${SNR}_{in}(f)$ and ${SNR}_{out}(f)$ are input SNR and output SNR, respectively. ${DQE}_{value}(f)$ expresses the SNR transfer characteristics in X-ray imaging systems according to the f [7]. The SNR and DQE improve as the conversion efficiency between each component in the imaging system increases, and they are currently the most actively studied fields in radiation image detection systems.

Digital radiography (DR) systems are most commonly used in various fields, such as medical diagnosis, nondestructive testing, and sample analysis, owing to their convenience for real-time inspection, wide dynamic range, and improvement of physical limitations on storage space compared to film and computed radiography (CR) systems [8]. Recently, DR systems have been required to use lighter and thicker scintillators to reduce patient exposure [9]. The fact that the diagnosis should be made while maximally reducing patient exposure according to the “as low as reasonably achievable” (ALARA) principle [10] contributes to the consideration of the use of thick scintillators so that the information of the detected radiation can be significantly reflected in the actual image. However, an increase in the scintillator thickness has a trade-off relationship in terms of reducing the image resolution [11]. As the scintillator thickness increases, the amount of light relative to the detected radiation increases. However, blurring due to light scattering also increases, thereby reducing the resolution of the image detection system [12]. This is a major cause of difficulty for readers in determining the fine structure of an object. In addition, the latest techniques based on radiographic images, such as microstructure production using 3D printing, are important because the resolution of the imaging system determines the production accuracy [13]. Noise is inevitable in radiographic systems requiring thin scintillators.

Various noise reduction methods have been introduced to overcome the physical limitations of radiation image detection systems in software applications. Traditional filtering-based noise reduction methods include average and median filters [14]. The average filter is primarily used for Gaussian and uniform noise, whereas the median filter is generally known as an effective filter for salt-and-pepper noise. Various noise reduction algorithms exist, such as gradient models [15,16] and non-local self-similarity models [17]. These methods are widely used as effective filtering methods for noise cancellation while preserving edge structures. A method of removing noise components by applying threshold values to high-frequency regions in other domains (e.g., Fourier and wavelet domains) has also been introduced. Wang et al. implemented a noise-reduction algorithm using a wavelet transform on a medical X-ray image [18]. They proposed a new type of directional adaptive median filter to reduce the noise amplitude in the wavelet domain by considering Poisson and Gaussian noise. Lee et al. investigated noise analysis and estimation in the non-subsampled contourlet transform (NSCT) domain, which represents an effective multi-resolution analysis for scale and direction decomposition [19]. Recently, machine learning using vast amounts of data has been extensively studied because computer hardware has gradually improved, and the development of graphics processing units (GPUs) has reduced calculation time and memory constraints. Studies on noise reduction have been actively conducted, including those based on convolutional neural networks (CNNs) [20,21]. The CNN-based noise reduction method showed outstanding results when the feature maps were well extracted using the convolution layer and the threshold was well operated through data-driven training with a fine-tuned dataset. Kang et al. performed noise reduction in low-dose X-ray computed tomography (CT) using a deep CNN architecture with directional wavelets [22]. Sun et al. investigated noise reduction using a generative adversarial network (GAN) for DR [23]. Despite the introduction of these effective noise reduction methods, some limitations remain. First, the level of noise reduction must be determined. Many studies have derived noise-reduction results, and quantitative evaluation factors have been presented. However, an increase in quantitative evaluation factor values, such as the SNR and peak SNR (PSNR), does not indicate an absolute improvement in comprehensive image quality [24]. This means that the parameters of noise reduction methods should be optimized based on reasonable image quality goals [25]. Second, noise reduction is necessary when considering various features, such as resolution and contrast. The resolution degrades through an excessive smoothing process, and clinically meaningful information can be removed [26]. In addition, reducing fluctuations in low-contrast lesions can change the contrast between target regions, which should be considered as a factor that significantly affects the accuracy of the diagnosis [27].

Therefore, we investigated an optimization method for noise reduction parameters to restore images that consider both resolution and contrast while obtaining noise characteristics similar to those obtained using a thick scintillator. The proposed method derives the optimal parameters of noise reduction methods that provide the best perceptual quality between radiographic images taken with thin and thick detectors with the same geometry. The viability of the proposed method was verified experimentally. The following sections briefly describe the proposed framework and discuss its qualitative and quantitative results.

## 2. Materials and Methods

### 2.1. Normalized Noise Power Spectrum (NNPS) Depending on Scintillator Thickness

To evaluate the noise performance measured with a detector in a radiological imaging system, the noise power spectrum (NPS) is often measured [28,29]. NPS representation is standard and useful for understanding the noise characteristics of an imaging system. There are two main approaches to measuring the NPS: indirect, using an auto-covariance function, or direct, by smoothing periodograms [30,31]. The NPS is calculated by sampling the region of interest (ROI) and calculating the periodograms through the discrete Fourier transform. The relationship between the sample size and the subimage has been extensively studied as it determines the accuracy of the NPS estimate [32,33].

**Figure 1:**
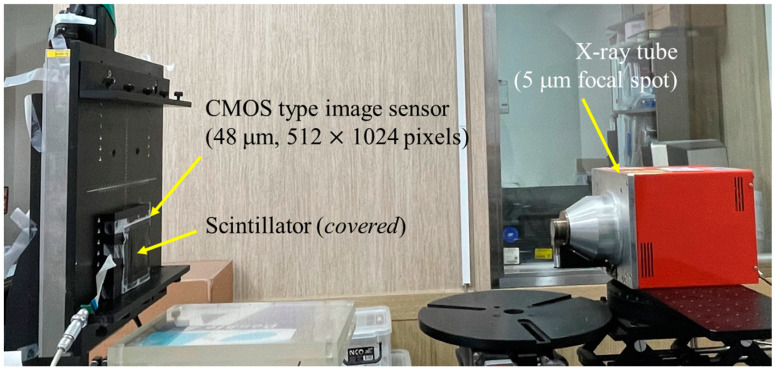
Photographs of the radiography imaging system, which consists of an X-ray tube (L10321, Hamamatsu Photonics K.K., Japan focal spot size: 5 μm) and a CMOS type image sensor (Rad-icon, pixel size: 48 μm, pixel matrix: 512 × 1024 pixels, and analog-to-digital conversion resolution: 16 bit) used in the experiment. CMOS, complementary metal–oxide–semiconductor.

The IEC 62220-1-1:2015 standard for radiographic image detectors overlaps many ROIs and subimages to increase the number of samples and improve measurement precision. It also uses a radial averaging technique with no loss of frequency resolution through directional NPS measurements along each axis [33,34,35,36]. To observe the improvement in noise characteristics using the proposed method, we acquired radiographic images using detectors with thin and thick scintillators according to RQA-5 conditions, which are general radiographic acquisition conditions. The radiation exposure conditions were as follows: source-to-detector distance (SDD), 150 cm; tube voltage, 70 kV; and additional filter, 21.0 mm Al. The X-ray tube (L10321, Hamamatsu Photonics K.K., Japan; focal spot size: 5 μm) and Gd_2_O_2_S:Tb scintillator-based complementary metal–oxide–semiconductor (CMOS) image sensor (pixel size: 48 μm, pixel matrix: 512 × 1024 pixels, and analog-to-digital conversion resolution: 16 bits) were used in this study. The experimental setup is shown in Figure 1.

**Figure 2:**
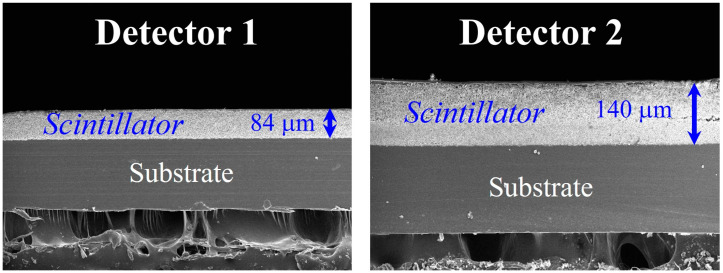
Scanning electron microscopy (SEM) images of each scintillator thickness: thin scintillator (detector 1) was 84 μm, and thick scintillator (detector 2) was 140 μm.

Figure 2 presents the scanning electron microscopy (SEM) images of the thin scintillator (detector 1) and the thick scintillator (detector 2). The thickness of detector 1 was 96 μm, and that of detector 2 was 140 μm. We manually replaced the scintillator in front of the CMOS sensor to obtain radiographic images without changing the imaging system or the photographed object. Radiographic images were acquired under RQA-5 exposure conditions in the absence of objects.

**Figure 3:**
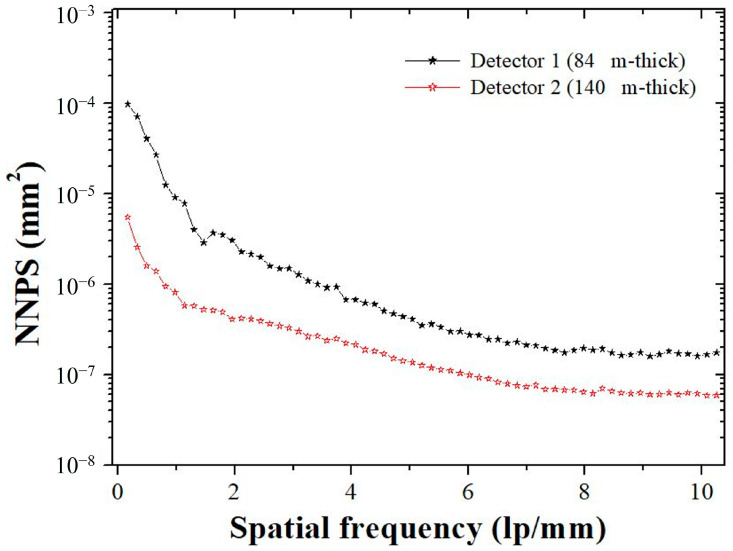
A 1D plot of the normalized noise power spectrum (NNPS) of two detectors according to the IEC 62220-1-1:2015 RQA-5 protocol.

Figure 3 shows the normalized NPS (NNPS) of the thin and thick scintillator-based detectors. The 2D NNPS were calculated using Equation (4):

(4) $$NNPS\left(f_{u},f_{v}\right)=\frac{\sum_{m=1}^{M}\langle {\left|F\{I\left(x_{l},y_{m}\right)-S\left(x_{l},y_{m}\right)\}\right|}^{2}\rangle }{{\left(\bar{S}\left(x,y\right)\right)}^{2}},$$

where $I\left(x_{l},y_{m}\right)$ and $S\left(x_{l},y_{m}\right)$ are the average ROIs of *l*th and *m*th, respectively. It can be assumed that a flat-intensity area is generated that retains only the noise component while subtracting these average ROIs. $\bar{S}\left(x,y\right)$ is the ensemble-averaged signal of the white images, which were obtained repeatedly. The 1D NNPS was calculated according to the spatial frequency represented by axial and radial averages using several frequency bins from the 2D NNPS, with $f_{u}$ and $f_{v}$ as the coordinate system [37].

### 2.2. Proposed Noise Reduction Framework Based on the Optimal Parameter Estimation

Among the various noise reduction algorithms mentioned above, the non-local means (NLM) algorithm is known as the leading method for reducing noise while preserving edges [38,39]. The NLM algorithm has been applied to various medical images such as magnetic resonance imaging (MRI) [40], ultrasound [41], positron emission tomography (PET) [42], and single photon emission computed tomography (SPECT) [43] as well as X-ray or CT [25,44] images to prove its effectiveness, and it is applied as a regularization model to improve the performance of various image processing methods, including deblurring, inpainting, and reconstruction [45,46,47]. The NLM algorithm is defined as follows:

(5) $$NL\left(I(i)\right)=\sum_{j}w\left(i,j\right)I(i),$$

(6) $$w\left(i,j\right)=\frac{1}{Z(i)}exp\left(-\frac{{\left\|p_{i}-p_{j}\right\|}_{2}^{2}}{h^{2}}\right),\;\sum_{j}w\left(i,j\right)=1,\;0\leq w\left(i,j\right)\leq 1,$$

(7) $$Z\left(i\right)=\sum_{j}exp\left(-\frac{{\left\|p_{i}-p_{j}\right\|}_{2}^{2}}{h^{2}}\right),\;$$

where I(i) denotes the pixel for noise reduction corresponding to the region adjacent to i; $w\left(i,j\right)$ is the Euclidean distance weight with a similar pattern; and $p_{i}$ and $p_{j}$ are square kernels for using the search window centered on i and j. The wider the search window, the better the image quality because more areas were compared. $w\left(i,j\right)$ can have a value between zero and less than one and must sum to one. Thus, the NLM algorithm attempts a noise reduction approach by searching for regions in the image that have a similar pattern to the surrounding regions and then implementing an average of these regions [48,49]. This method improves the image quality because it removes noise through patch-based similarity measurement rather than the pixel-based similarity measurement of existing filters, including the Gaussian filter, anisotropic filter, total variation filter, and Yaroslavsky neighborhood filter [50]. However, the patch-based noise reduction method has a major drawback in terms of computational cost. To overcome this problem, the calculation approach was improved with vectorization when calculating the weight function $w\left(i,j\right)$ [51]. Accelerated NLM was performed in this study, and a patch and window size of 2 × 2 were empirically used. The processing time of accelerated NLM in an image of 512 × 1024 pixels is approximately 0.47 s; it was about 8 times faster than conventional NLM. The parameter that should be noted when performing NLM is h. Here, h is sigma value, which controls the smoothing rate in the NLM algorithm. Increasing the h value reduces the noise component but also reduces sharpness. Noise reduction should be considered in conjunction with avoiding resolution degradation.

Therefore, the purpose of this study is to propose a method for predicting the optimal smoothing parameter h, which allows the image obtained from a thin scintillator to maintain sharpness as much as possible while reducing the noise component of the image obtained from the thick scintillator. To derive the optimal parameters, the edge preservation index (EPI), which quantitatively analyzes the sharpness of the image based on the reference, and the signal-to-noise ratio (SNR), which indicates the characteristics of noise compared to the signal, were used. These were calculated as follows [25,52]:

(8) $$EPI=\frac{\Gamma \left(\Delta p_{1}-\bar{\Delta p_{1}},\Delta p_{2}-\bar{\Delta p_{2}}\right)}{\sqrt{\left(\Delta p_{1}-\bar{\Delta p_{1}},\Delta p_{1}-\bar{\Delta p_{1}}\right)}\circ \Gamma \left(\Delta p_{2}-\bar{\Delta p_{2}},\Delta p_{2}-\bar{\Delta p_{2}}\right)},\;\Gamma \left(a,b\right)={\sum_{i,j\in ROI}a\left(i,j\right)b\left(i,j\right)}^{2},$$

(9) $$SNR=\frac{mean}{std},$$

where $p_{1}$ and $p_{2}$ represent the reference and measured images, respectively. $\bar{\Delta p}$ operates the Laplacian filtering in the ROI. This was used as a reference for the image of a thin scintillator. It can be assumed that the closer the EPI value is to one, the better the sharpness of the image is maintained compared to the reference. SNR can be defined as the mean value divided by the standard deviation in ROI. Generally, as the value of the NLM smoothing parameter increases, the SNR increases. By reflecting the characteristics of the two quantitative evaluation factors, the changes in the EPI and SNR values were tracked as the values of the smoothing parameter increased, and an algorithm was proposed by setting the point where the two values intersected as the optimal value of the smoothing parameter.

**Figure 4:**
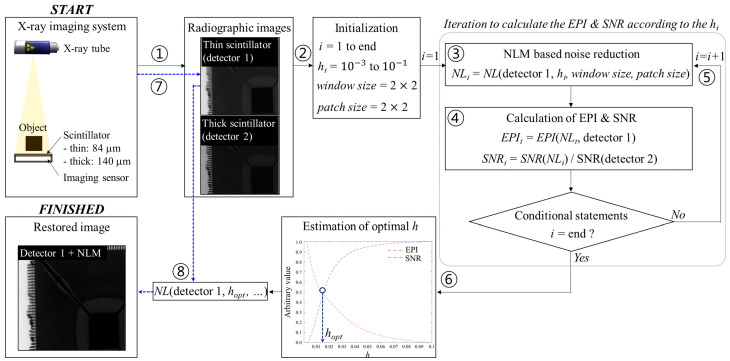
Schematic framework of proposed optimization scheme for adaptive noise reduction in radiography. Abbreviations: EPI, edge preservation index; NLM, non-local means; SNR, signal-to-noise ratio.

Figure 4 shows a brief framework of the process for predicting the optimal smoothing parameters using the NLM method. In brief, ① thin scintillator (detector 1)- and thick scintillator (detector 2)-based radiographic images obtain the established X-ray imaging system. Then, ② the following variables are initialized to perform the NLM algorithm: *i* is the iteration number, $h_{i}$ denotes the smoothing factor in Equations (6) and (7), the window size is an integer indicating the size of the search region, and the patch size is an integer indicating the neighborhood size for weight computation. Here, $h_{i}$ is empirically set to 100 steps from 10^−3^ to to 10^−1^. ③ The noise reduction is performed based on the NLM algorithm using the detector 1 image. EPI and SNR are computed. Here, the EPI is calculated by comparing the NLM result (*NL_i_*) obtained with the $h_{i}$ and detector 1 images for reference. *SNR_i_* normalizes the SNR result of *NL_i_* to that of the detector 2 image owing to the equalization of the scale to the EPI. ⑤ After performing the framework by repeating until the end of i, ⑥ the EPI and SNR values according to the *h_i_* are expressed in a graph. It is noteworthy that the section in which the two factors intersect is selected as the case in which the sharpness and noise characteristics are most appropriately improved in the noise reduction image, and $h_{i}$ is set to the optimal smoothing parameter. Finally, ⑦ a radiographic image is taken on a thin scintillator, and then ⑧ it is possible to restore the optimal image based on the pre-calculated adaptive parameter.

### 2.3. Materials

**Figure 5:**
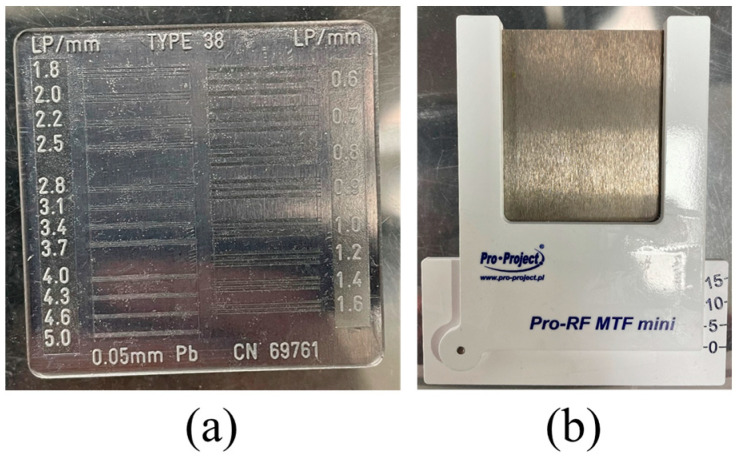
(**a**) A high-resolution line chart phantom and (**b**) an edge test device according to IEC 62220-1 in the experiment.

Figure 5 shows (a) a high-resolution line chart phantom (Type 38, CN 69761, Active Radsys, Italy) and (b) an edge test device (Pro-RF MTF mini, 02-127, DIAGNOMATIC, Poland) used in the experiment. The high-resolution patterns of the line chart phantom had 20 groups from 0.6 to 5.0 lp/mm. The edge-test device consisted of a tungsten plate fixed on a lead plate according to the IEC 62220-1 protocol. The radiographic image was obtained by tilting the edge device at least 0 to 3° to avoid potential aliasing that may occur during the process of obtaining the modulation transfer function (MTF) [53]. To perform noise reduction, a normal workstation was used with OS Windows 10, CPU Intel Core i7 10700 @ 2.90 GHz, and RAM 64 GB.

### 2.4. Quantitative Evaluation of Image Quality

The NNPS and MTF were used to quantitatively evaluate the acquired images. Donini et al. introduced analysis software for radiographic imaging systems, which was programmed in Java, a proven free program that supports the ImageJ plugin [54]. Two factors were measured using this program, and pre-processing, such as dark analysis, image lag analysis, and a uniform test, was performed.

Additionally, a blind/referenceless image spatial quality evaluator (BRISQUE), a representative nonreference-based parameter, was used to comprehensively evaluate the noise level and spatial resolution [55]. The BRISQUE function provided via MATLAB (R2021a, MathWorks, Natick, MA, USA) was used for quantitative image evaluation.

## 3. Results and Discussion

**Figure 6:**
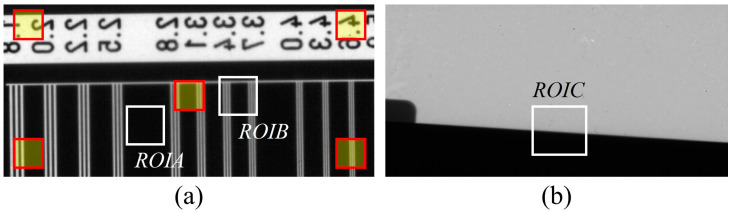
Sample images for ROI setting: (**a**) ROIA and ROIB for evaluating enlarged images and the red box ROI set for SNR calculation; (**b**) ROIC marked for MTF measurement in edge phantom images. Abbreviations: MTF, modulation transfer function; ROI, region of interest; SNR, signal-to-noise ratio.

Figure 6 shows the ROI set in the sample image. Figure 6a shows ROIA and ROIB for magnification in the resolution phantom image, and the ROIs are set in the red box for SNR calculation. Here, the ROIA can be used to observe the amount of noise contained in a homogeneous area. The ROIB was selected to determine how much sharpness was maintained. Figure 6b shows the ROIC area for the MTF measurement in the edge phantom image. 

**Figure 7:**
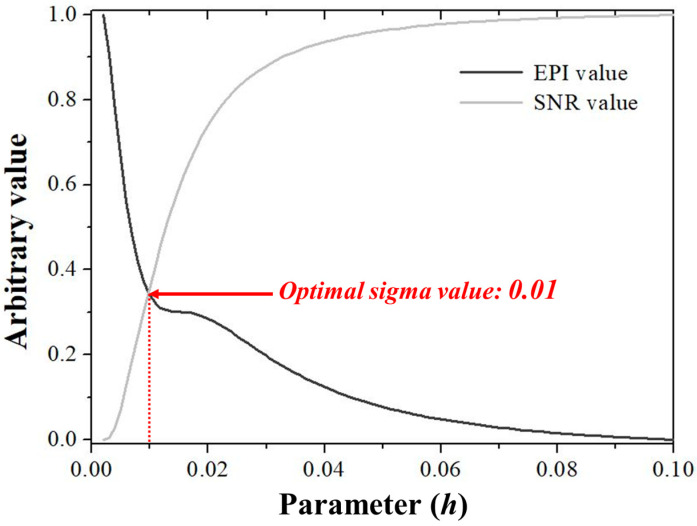
Graphs of EPI and SNR values with respect to the sigma value for the NLM noise reduction algorithm. The two graphs overlapped when the sigma value was 0.01. Abbreviations: EPI, edge preservation index; SNR, signal-to-noise ratio.

Figure 7 shows a graph deriving the EPI and SNR values according to the sigma value of the NLM noise reduction algorithm. As the sigma value of the NLM algorithm increased, the EPI tended to decrease, and the SNR improved. The optimal sigma value derived in this study was set at the point at which the graphs of the two quantitative evaluations coincided. The optimal sigma value of the NLM noise reduction algorithm for the X-ray detector imaging system used in this study was confirmed to be 0.01. Based on these results, five detector systems were used to acquire the resulting images. In addition to detectors 1 and 2 described in Section 2.1, the NLM noise reduction algorithm was applied to images acquired from a relatively thin detector system. The NLM algorithm used sigma values of 0.005, 0.05, and 0.01 for detector 1. Additionally, the reason for comparing the sigma values of 0.005 and 0.05 is to investigate the quantitative results of considering more on the sharpness aspect or vice versa compared to the 0.01 sigma value.

**Figure 8:**
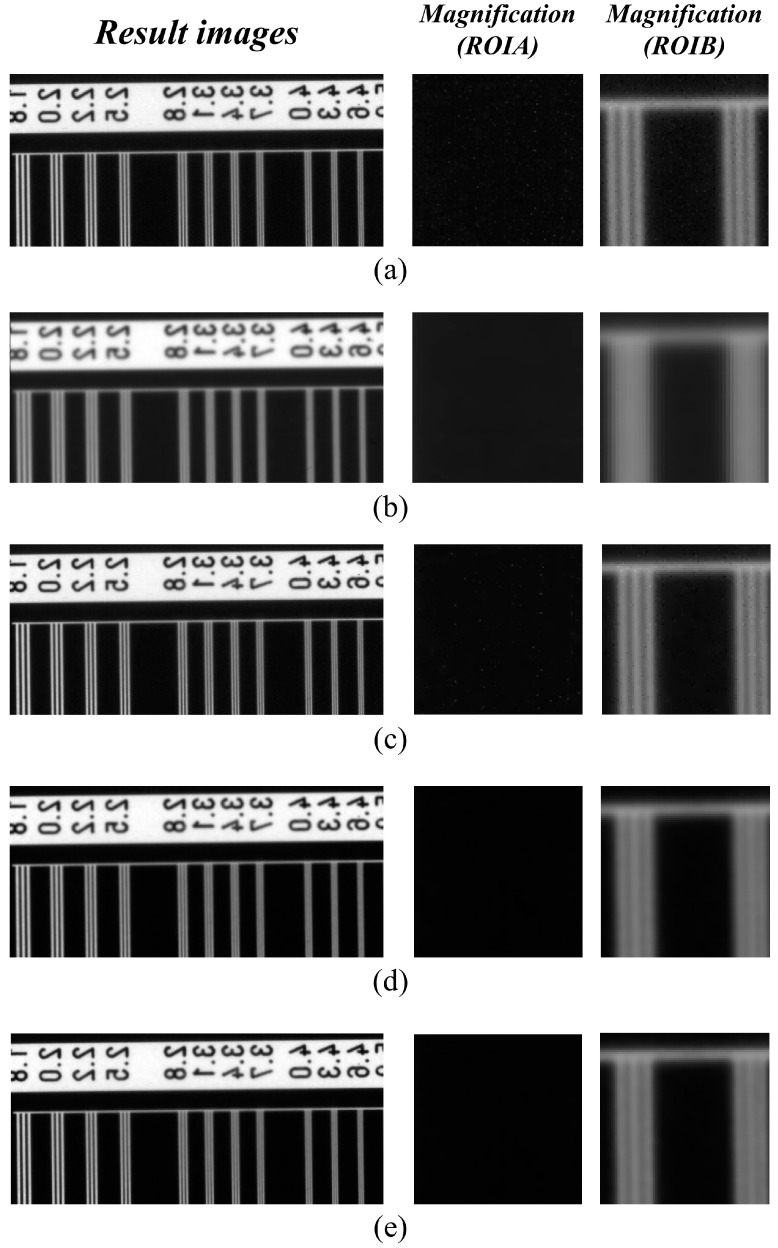
High-resolution line chart phantom result images derived by applying each detector system and NLM noise reduction algorithm. In all systems, the resolution phantom image and two enlarged ROI images were expressed. (**a**,**b**) are the resulting images obtained from detectors 1 and 2, respectively. (**c**–**e**) are the resulting images of applying the NLM noise reduction algorithm’s sigma values of 0.005, 0.05, and 0.01 to detector 1, respectively. Abbreviations: NLM, non-local means; ROI, region of interest.

Figure 8 shows the resulting image of the high-resolution line-chart phantom as a function of the detector system. Figure 8a,b show the resulting images obtained from thin detector 1 and thick detector 2, respectively. In the image observed by enlarging the ROIA and ROIB areas in Figure 6a, we observed that the noise level of detector 1 increased, and blurring was applied to the image acquired with detector 2. The images obtained by applying the NLM noise reduction algorithm to the images acquired with detector 1 are shown in Figure 8c–e. Figure 8c shows the result of applying the NLM algorithm to the X-ray image using a sigma value of 0.005, confirming that the noise level decreased compared to detector 1. In addition, as shown in Figure 8d, we confirmed that when the sigma value of the NLM algorithm was applied to the highest level, the noise level was greatly reduced and blurring increased relative to the image (Figure 8c). As a result, we proved that the optimal value for the noise level and blurring in the image was obtained using the NLM algorithm based on the sigma value of 0.01 proposed in this study (Figure 8e).

**Figure 9:**
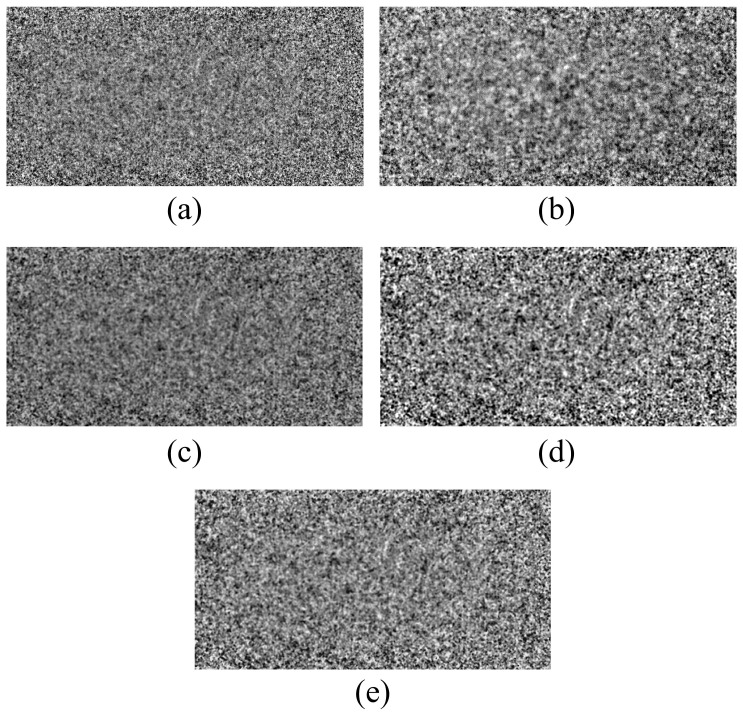
White image results for NNPS measurement: (**a**,**b**) are the resulting images obtained from detectors 1 and 2, respectively. (**c**–**e**) are the resulting images of applying the NLM noise reduction algorithm’s sigma values of 0.005, 0.05, and 0.01 to detector 1, respectively. Abbreviations: NLM, non-local means; NNPS, normalized noise power spectrum.

**Figure 10:**
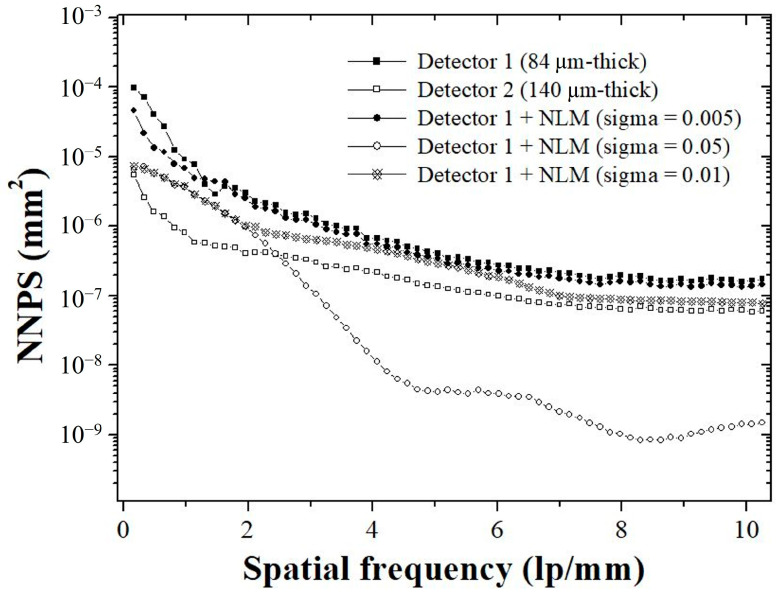
Graphs of NNPS values for different detector systems. The lowest NNPS value was derived from the NLM algorithm using the highest sigma value, and the proposed system was confirmed to have superior values compared to detector 1. Abbreviations: NLM, non-local means; NNPS, normalized noise power spectrum.

Figure 9 shows the white image of each detector system used to derive the NNPS results. The NNPS results derived from Figure 9 are shown in Figure 10. All detector systems showed a tendency for the NNPS data to decrease as the spatial frequency increased. The detector 2 system, which had a relatively higher thickness, had lower NNPS values than the detector 1 system. This is consistent with the theory that noise level decreases as detector thickness increases. The lowest NNPS result was obtained when the sigma value of the NLM noise reduction algorithm had a large value of 0.05. When only considering the distribution of noise according to the spatial frequency, we confirmed that it may be advantageous to increase the sigma value of the NLM algorithm and apply it to X-rays. The NNPS results of the NLM noise reduction algorithm, modeled by setting the sigma value proposed in this study to 0.01, showed almost similar trends to detector 2. When comprehensively analyzing the NNPS results, we proved that the NLM algorithm applying the proposed 0.01 sigma value can obtain a noise level similar to that of images using a relatively thick X-ray detector.

**Figure 11:**
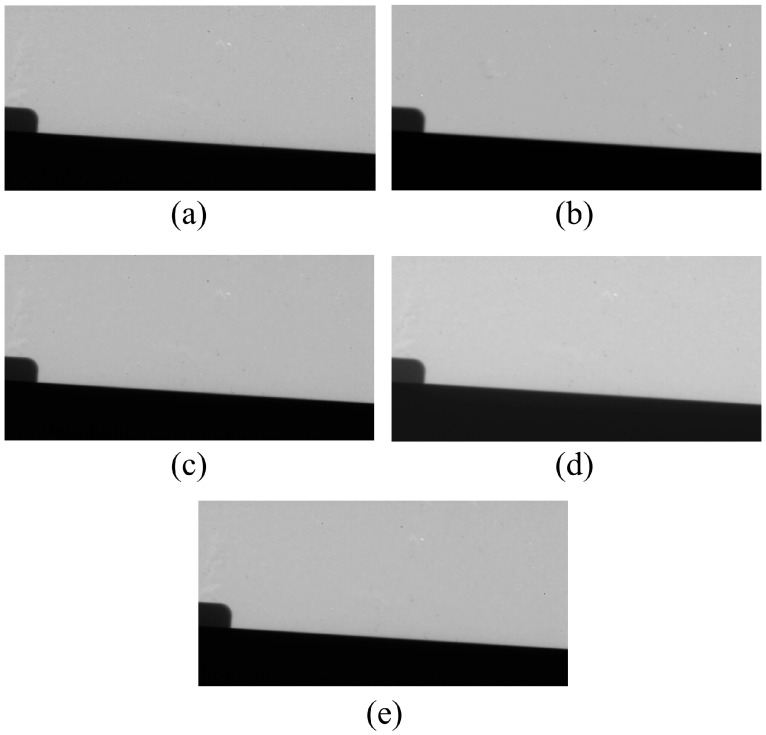
Edge phantom image derived by applying each detector system and NLM noise reduction algorithm. (**a**,**b**) are the resulting images obtained from detectors 1 and 2, respectively. (**c**–**e**) are the resulting images of applying the NLM noise reduction algorithm’s sigma values of 0.005, 0.05, and 0.01 to detector 1, respectively. NLM, non-local means.

**Figure 12:**
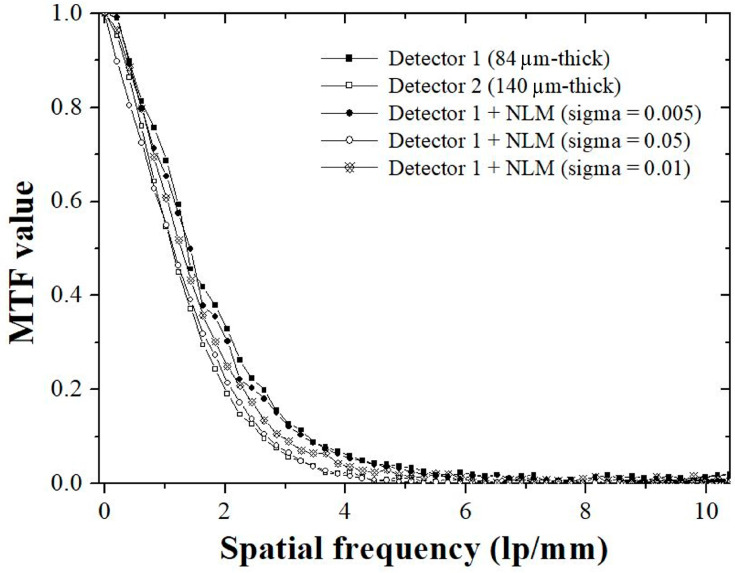
Graphs of MTF values for different detector systems. The highest MTF value was derived from the NLM algorithm using the lowest sigma value, and the proposed system was confirmed to have superior values compared to detector 2. Abbreviations: MTF, modulation transfer function; NLM, non-local means.

However, although the noise level can be significantly reduced when the highest sigma value of the NLM algorithm is applied to detector 1, increased blurring inevitably occurs. Figure 11 shows the resulting images from various detector systems acquired using edge phantom images. The MTF graph in ROIC, shown in Figure 6b, is shown in Figure 12. The MTF values decreased with increasing spatial frequency for all detector systems. The MTF results showed the best values for detector 1, which was the thinnest detector system. Conversely, the lowest MTF results were obtained for the thickest system, detector 2. The MTF result of the NLM noise reduction algorithm modeled by setting the proposed sigma value to 0.01 resulted in a slightly lower value compared to when the value was set relatively small; however, an improved value was measured compared to detector 2. The 10% MTF value used to evaluate the spatial resolution was confirmed to be approximately 3.42 lp/mm in the NLM algorithm using the proposed sigma value of 0.01. An MTF value greater than 10% was confirmed to be approximately 13% higher than that of detector 2.

**Figure 13:**
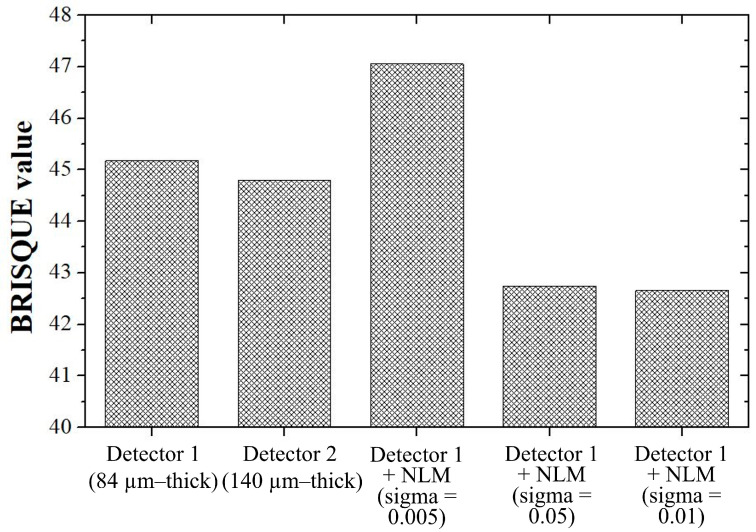
Graphs of BRISQUE values with respect to the detector systems. An excellent BRISQUE value was derived from the NLM algorithm using the proposed sigma value (0.01). The lowest BRISQUE value is obtained when the NLM algorithm with the highest sigma value is used. Abbreviations: BRISQUE, blind/referenceless image spatial quality evaluator; NLM, non-local means.

The NNPS and MTF calculated using the proposed method (the NLM algorithm using a 0.01 sigma value) have the advantage of being able to appropriately control the noise amplification of thin detectors and the blurring of images of thick detectors. To evaluate the noise level and degree of blurring comprehensively, a no-reference-based parameter was used. The BRISQUE evaluation parameter indicates that the smaller the value, the more similar the image to be measured is to an ideal image. Figure 13 shows a graph of the BRISQUE values according to the detector system. The BRISQUE value measured in this study was approximately 42.66 when the proposed NLM algorithm was applied to the image acquired with detector 1. The BRISQUE value was confirmed to be the best among the detector systems used in this study, and we proved that the proposed method improved by 5.57 and 4.76% compared with detectors 1 and 2, respectively.

Consequently, the NLM algorithm applying the proposed optimized sigma value yielded excellent quantitative evaluation data for an imaging system based on a thin X-ray detector. As a result of the NNPS, the X-ray image obtained using the proposed method was found to be similar to the value obtained in a system with a relatively thick detector. In addition, in the MTF graph, the proposed method showed spatial resolution values similar to those of the X-ray images obtained using a thin scintillation-based detector. Although the best value was not derived from the system being compared for the NNPS and MTF results, the BRISQUE value, which comprehensively evaluates the noise level and degree of blurring, showed that the proposed method provided superior data. The BRISQUE evaluation factor proposed by Mittal et al. is a representative method for analyzing naturalness in the spatial domain and shows statistically superior characteristics compared to PSNR [55]. Therefore, the BRISQUE evaluation factor is widely used when evaluating noise levels and blurring in medical images, and its applicability in image quality analysis of X-ray images has also been partially reported [56].

The proposed method provides optimal parameters to improve the comprehensive image quality characteristics of the NLM algorithm; however, there are still several issues to consider. First, the proposed method performs parameter optimization in a single domain. Among the methods for predicting noise levels, the most actively used is multi-resolution analysis (MRA), which includes the wavelet [57], NSCT [19], and non-subsampled shearlet transform (NSST) domains [58]. Domain-transformation-based MRA has a significant advantage because accurate noise level estimation is advantageous for each sub-band. The proposed method reduced the noise component of each sub-band image using the MRA method, and the effect of improving the image quality was confirmed. However, when noise reduction is performed on each sub-band image, artifacts such as ring artifacts [59] and pseudo-Gibbs artifacts occur [60], and we plan to correct them and conduct comprehensive analysis studies on them in the future. Second, this is the case where there are various parameters in the noise reduction algorithm. For NLM algorithms, three main parameters—patch size, window size, and smoothing factor h—must be considered. The patch size did not significantly affect the smoothing of the image, and a window size of 2 × 2 pixels is used among 1 × 1 pixels to 4 × 4 pixels, owing to a serious loss of resolution, except for 2 × 2 pixels. Therefore, it is possible to set the window and patch sizes constantly and find the optimal h. However, if two or more parameters are to be considered in the noise reduction algorithm, it may be difficult to directly apply the proposed method. Quantitative optimization of multiple parameters is expected to be performed through deep learning, including the approach of He et al. [61]. However, systematic processes, such as the reliability of label data and standardization of data construction, should be performed. Finally, it is a criterion for determining the optimal parameters for the imaging system. Because the loss of spatial resolution accompanying noise reduction leads to the loss of low-contrast lesions [27], noise reduction can be performed under optimal conditions, as in the proposed method. However, the criteria for the optimal parameters of the noise reduction algorithm may differ depending on the imaging system and radiation exposure conditions. For instance, the overall image quality and lesion classification accuracy can be improved if noise reduction is performed to emphasize the SNR in gamma-ray-based images with high quantum noise [62,63]. Conversely, in the case of microscopic images that provide a large number of photons, high-definition image restoration can be performed by considering noise reduction and resolution improvement [64]. In such cases, optimal noise parameters should be considered within a certain window. By selecting a parameter included in a certain window from the optimal parameter derived using the proposed method, an image with more emphasis on sharpness or an image with more emphasis on SNR can be provided according to the shooting conditions and purpose. In this regard, a comprehensive verification of various applications will be conducted in future studies.

Appropriate noise reduction has positive effects in various fields of application research based on radiation data. Data-based regression or classification, such as machine learning, plays an important role in noise filtering during pre-processing [65]. This helps make accurate predictions by removing obstacles. Pre-processing of images contributes to improving the accuracy of extracting features from images [66], and the proposed method is expected to contribute significantly to feature extraction using various images through optimal noise removal suitable for each imaging system. In addition, it is expected that the proposed method for generating label data, considering both sharpness and noise characteristics, will be applied very effectively for machine learning. Based on this approach, it can contribute to improving image performance through quantified dataset construction and sophisticated model training.

Currently, 3D printing technology is being actively researched in the medical field, and the proposed algorithm can play a role in pre-processing, such as edge detection and segmentation, when used as the dataset for this technology [67,68,69,70]. The quality of 3D printing models depends on the direct impact on radiographic image quality [71], and studies have been conducted to investigate the level at which 3D printing is possible even at low or high radiation doses to remove noise components [72]. In particular, there is a need in the medical field to perform 3D printing based on low-dose CT data because the patient dose is reduced as much as possible. Noise filtering is mainly used for pre-processing as a complement to low-dose CT images and for research on the verification and optimization of 3D printing results according to various variables, including the noise filtering method and kernel size [73]. The proposed method can provide quantified noise-reduction radiation data. Therefore, it is expected that 3D printing can improve production reproducibility.

## 4. Conclusions

In this study, the proposed framework optimizes the adaptive parameters in the noise reduction method to produce a balanced result between resolution and SNR. Compensating for the low noise characteristics of thick-scintillator-based images while preserving high resolution was proposed. Quantitative evaluation results show that the proposed approach can effectively improve the image quality of thin-scintillator-based images. Consequently, the proposed method is expected to be considerably effective in providing optimized parameters that can maximize the image quality characteristics while maintaining sharpness when using noise reduction algorithms. In addition, this method is expected to contribute to the standardization of pre-processing parameters when building training datasets for machine learning. This will contribute significantly to improving the training performance.

## Data Availability

Data are contained within the article.
